# Association between single nucleotide polymorphisms in the antioxidant genes *CAT*, *GR* and *SOD1*, erythrocyte enzyme activities, dietary and life style factors and breast cancer risk in a Danish, prospective cohort study

**DOI:** 10.18632/oncotarget.18062

**Published:** 2017-05-22

**Authors:** Tine Iskov Kopp, Ulla Vogel, Lars Ove Dragsted, Anne Tjonneland, Gitte Ravn-Haren

**Affiliations:** ^1^ Research Centre for Prevention and Health, Glostrup, Denmark; ^2^ Danish Cancer Society Research Center, Copenhagen, Denmark; ^3^ National Food Institute, Technical University of Denmark, Søborg, Denmark; ^4^ National Research Centre for the Working Environment, Copenhagen, Denmark; ^5^ Department of Nutrition, Exercise and Sports, University of Copenhagen, Copenhagen, Denmark

**Keywords:** breast cancer, prospective cohort study, single nucleotide polymorphisms, gene-environment interactions, antioxidant enzymes

## Abstract

Exposure to estrogens and alcohol consumption - the two only well-established risk factors for breast cancer - are capable of causing oxidative stress, which has been linked to progression of breast cancer. Here, five functional polymorphisms in the antioxidant genes *SOD1*, *CAT* and *GSR* were investigated in 703 breast cancer case-control pairs in the Danish, prospective “Diet, Cancer and Health” cohort together with gene-environment interactions between the polymorphisms, enzyme activities and intake of fruits and vegetables, alcohol and smoking in relation to breast cancer risk. Our results showed that genetically determined variations in the antioxidant enzyme activities of *SOD1*, *CAT* and *GSR* were not associated with risk of breast cancer *per se*. However, intake of alcohol, fruit and vegetables, and smoking status interacted with some of the polymorphisms in relation to breast cancer risk. Four polymorphisms were strongly associated with enzyme activity, but there was no interaction between any of the studied environmental factors and the polymorphisms in relation to enzyme activity. Additionally, single measurement of enzyme activity at entry to the cohort was not associated with risk of breast cancer. Our results therefore suggest that the antioxidant enzyme activities studied here are not major determinants of breast cancer risk.

## INTRODUCTION

Breast cancer (BC) is the most common type of cancer in Denmark among women and the second leading cause of cancer death after lung cancer [1]. The incidence has increased during the last decade [1], which warrants further knowledge about the underlying mechanisms. Environmental factors, in interplay with genetics, are known to be important in the etiology of BC [2–5]. Therefore, examining gene-environment interactions is an effective tool for identifying biological pathways in disease etiology.

The only well-established risk factors for BC are associated with prolonged exposure to estrogens [6,7] and alcohol consumption [8–10]. Both exposure to estrogens and alcohol consumption are capable of causing oxidative stress [reviewed in 11], which has also been linked to progression of cancer, including BC [12–18]. Oxidative stress is caused by an imbalance between the generation of reactive oxygen species (ROS), such as superoxide anions, hydrogen peroxide and hydroxyl radicals, and the antioxidant defense, including various enzymes and high and low molecular weight antioxidants (including glutathione (GSH)), leading to DNA damage, lipid peroxidation and protein oxidation. An efficient antioxidant defense will limit oxidative damage by ensuring a reducing environment [19,20]. The enzymatic antioxidant defense system is complex and may be described as an interacting network of several enzymes, including glutathione reductase (GR), superoxide dismutase (SOD) and catalase (CAT). Where GR catalyzes the reduction of GSH, an important antioxidant, superoxide anion radicals are dismutated by SOD to hydrogen peroxide. CAT converts hydrogen peroxide to water and oxygen, thereby preventing formation of the highly reactive hydroxyl radical. BC may partly be caused by oxidative damage combined with a failure of antioxidants to protect the breast tissue [14]. Smoking and alcohol consumption are sources of ROS whereas fruit and vegetables are rich in dietary antioxidants. Furthermore, several studies have indicated that an increased intake of fruits and vegetables may affect the enzymatic antioxidant defense system, leading to increased enzyme activities [21–24]. These dietary and life style choices may modulate risk of BC by altering the level of oxidative stress, such that alcohol consumption and smoking increase the risk, while intake of fruit and vegetables decreases the risk. However, where meta-analyses have not provided consisting evidence of an inverse association between intake of fruit and vegetables and BC risk on one side [25–29], and positive association between smoking and BC risk on the other side [reviewed in 30], interactions between smoking, alcohol consumption, fruit and vegetable intake, and polymorphisms in antioxidant genes in relation to BC risk, have been reported [30–34]. Thus, genetically determined variations in the activities of enzymes that protect against or generate oxidative stress could modify associations between dietary antioxidants and exogenous sources of ROS, and BC risk, and thus explain some of the inconsistencies in the results from these studies. Additionally, lower antioxidant enzyme activities in BC patients compared to healthy controls have been reported in several studies [35–38]. Prospective cohort studies may reveal whether these lowered activities are a cause or consequence of the disease in combination with examination of dietary and life style factors and their interaction with the enzymes in relation to risk of disease.

Functional polymorphisms may impact the enzymatic activities leading to altered antioxidant defense and thereby affect disease risk and health. We have previously found associations between functional polymorphisms in *GPX1* and *GPX4* genes, erythrocyte GPX activity, alcohol intake, hormone replacement therapy (HRT) use and BC risk in the Danish “Diet, Cancer and Health” (DCH) cohort [31,39]. In the present study, we wanted to extend these findings with polymorphisms in the antioxidant genes *SOD1* (encoding SOD), *CAT* (encoding CAT) and *GSR* (encoding GR) in a study group of 975 postmenopausal women with BC and 975 matched controls nested within the DCH cohort; and search for gene-environment interactions between the polymorphisms, enzyme activities and intake of fruits and vegetables, alcohol and smoking in relation to BC risk.

## RESULTS

Baseline characteristics of BC cases and matched controls are presented in Table 1. Findings regarding the included risk factors have been reported previously for the whole DCH cohort, for a subset of the present study, and for the present study group [31,40–47]. Among controls, the genotype distribution of the polymorphisms did not deviate from Hardy-Weinberg equilibrium (results not shown). None of the polymorphisms were significantly associated with risk of BC *per se* (Table 2); however, a tendency towards increased risk among variant carriers of the *SOD1*/rs202445 polymorphism, was observed (*P*=0.06). Only *GSR*/rs1002149 interacted weakly with alcohol intake in relation to risk of BC such that variant T-carriers had a 24% increased risk of BC per 10 g alcohol/day (95% CI: 1.09-1.42), whereas wild-type GG-carriers did not display alcohol-related modification of BC risk (*P*-value for interaction (*P*_int_)= 0.048) (Table 3). We also found interaction between *SOD1*/rs202445 and intake of fruits and vegetables in relation to risk of BC (*P*_int_=0.016) (Table 4). Carriage of the variant G-allele of *SOD1*/rs202445 was associated with a 13% increased risk of BC (95% CI: 1.03-1.25), whereas wild-type AA-carriers showed no modified risk of BC per 100 g fruits and vegetables per day (Table 4). Smoking status interacted with the *CAT*/rs1001179 polymorphism in relation to risk of BC (*P*_int_=0.0015) (Table 5). Variant A-carriers of the polymorphism, who did not smoke, had a 41% increased risk of BC (95% CI: 1.07-1.87) compared to non-smoking wild-type GG-carriers (Table 5).

**Table 1:** Baseline characteristics of the DCH study participants by selected demographic and established BC risk factors

Variable	Cases		Controls		IRR^a^ (95% CI)
	n (%)	Median (5-95%)	n (%)	Median (5-95%)	
Women	703 (100)		703 (100)		
Age at inclusion, years		57 (51-64)		57 (51-64)	
School education Short Medium Long	206 (29) 353 (50) 144 (20)		250 (36) 313 (45) 122 (17)		1.0 (ref.) 1.19 (0.92-1.54) 1.27 (0.90-1.78)
Body mass index, kg/m^2^		25 (20-34)		25 (20-34)	1.02 (0.97-1.08)^b^
Nulliparous	104 (15)		82 (12)		1.02 (0.65-1.58)^c^
Number of births		2 (1-4)		2 (1-4)	0.92 (0.79-1.05)
Age at first birth, years		24 (18-31)		23 (18-32)	1.06 (0.91-1.24)^d^
Use of HRT, years^e^		6 (0.5-20)		5 (0.5-20)	1.00 (0.87-1.15)^f^
Abstainers	18 (3)		22 (3)		0.92 (0.47-1.79)^g^
Alcohol intake, g/day		11 (1-43)		9 (1-40)	1.11 (1.03-1.20)^h^
Present smokers	241 (34)		264 (38)		0.93 (0.73-1.19)
Total fruit and vegetable intake, g/day		361 (118-785)		349 (108-819)	1.02 (0.97-1.08)^i^
Benign breast disease	139 (20)		88 (13)		1.64 (1.22-2.20)

Values are expressed as medians (5th and 95th percentiles) or as fractions (%). IRR: incidence rate ratio.

^a^ The risk estimates for BC are mutually adjusted.

^b^ The risk is estimated per additional 2 kg/m^2^.

^c^ The risk is estimated for nulliparous versus one birth at age 35.

^d^ The risk is estimated per additional 5 years.

^e^ Among ever users of HRT.

^f^ The risk is estimated per additional 5-year of HRT use.

^g^ The risk for abstainers compared to the increment of 10 g alcohol per day.

^h^ Among drinkers, risk estimate is estimated for the increment of 10 g alcohol per day.

^i^ The risk is estimated per additional 100 g intake fruit and vegetables per day.

**Table 2:** IRR for BC in relation to the studied polymorphisms

Gene	SNP	n_cases_ (%) (n=703)	n_controls_ (%) (n=703)	IRR^a^ (95% CI)	IRR^b^ (95% CI)	P-value^c^
*CAT*	rs1001179 GG GA AA GA+AA	408 (58) 251 (36) 44 (6) 295 (42)	409 (58) 253 (36) 41 (6) 294 (42)	1.00 (ref.) 1.00 (0.81-1.25) 1.08 (0.69-1.70) 1.02 (0.83-1.25)	1.00 (ref.) 1.05 (0.87-1.32) 1.07 (0.67-1.70) 1.06 (0.85-1.31)	0.65 0.79 0.62
*CAT*	rs12270780 GG GA AA GA+AA	393 (56) 267 (38) 43 (6) 310 (44)	405 (58) 267 (38) 31 (4) 298 (42)	1.00 (ref.) 1.02 (0.82-1.27) 1.37 (0.85-2.19) 1.06 (0.86-1.31)	1.00 (ref.) 1.01 (0.80-1.26) 1.29 (0.80-2.09) 1.04 (0.84-1.29)	0.96 0.30 0.73
*CAT*	rs769217 CC CT TT CT+TT	454 (65) 222 (32) 27 (4) 249 (35)	460 (65) 211 (30) 32 (5) 243 (35)	1.00 (ref.) 1.06 (0.84-1.34) 0.87 (0.50-1.51) 1.04 (0.83-1.30)	1.00 (ref.) 1.06 (0.83-1.35) 0.97 (0.55-1.71) 1.05 (0.83-1.32)	0.64 0.91 0.69
*GSR*	rs1002149 GG GT TT GT+TT	480 (68) 201 (29) 22 (3) 223 (32)	452 (64) 225 (32) 26 (4) 251 (36)	1.00 (ref.) 0.85 (0.68-1.06) 0.80 (0.44-1.45) 0.84 (0.68-1.05)	1.00 (ref.) 0.86 (0.68-1.09) 0.84 (0.46-1.55) 0.86 (0.69-1.08)	0.21 0.58 0.19
*SOD1*	rs202445 AA AG GG AG+GG	460 (65) 214 (30) 29 (4) 243 (35)	490 (70) 193 (27) 20 (3) 213 (30)	1.00 (ref.) 1.18 (0.93-1.50) 1.56 (0.86-2.80) 1.22 (0.97-1.53)	1.00 (ref.) 1.22 (0.96-1.56) 1.55 (0.85-2.84) 1.25 (0.99-1.59)	0.11 0.15 0.06

IRR: incidence rate ratio.

^a^ Crude.

^b^ Adjusted for parous/nulliparous, number of births, age at first birth, length of school education (low, medium, high), duration of HRT use (years), BMI (kg/m^2^), previous benign breast disease and alcohol intake (10 g/day).

^c^ *P*-value for the adjusted risk estimates.

**Table 3:** IRR for BC in relation to the studied polymorphisms per increment of 10 g alcohol per day among current drinkers^a^

Gene	SNP	n_cases_ (%) (n=664)	n_controls_ (%) (n=664)	IRR^b^ (95% CI)	IRR^c^ (95% CI)	*P*-value^d^
*CAT*	rs1001179 GG GA+AA	382 (58) 282 (42)	388 (58) 276 (42)	1.16 (1.05-1.27) 1.09 (0.96-1.24)	1.15 (1.04-1.26) 1.07 (0.94-1.22)	0.43
*CAT*	rs12270780 GG GA+AA	375 (56) 289 (44)	384 (58) 280 (42)	1.12 (1.02-1.23) 1.15 (1.02-1.30)	1.11 (1.00-1.22) 1.13 (1.00-1.28)	0.77
*CAT*	rs769217 CC CT+TT	430 (65) 234 (35)	434 (65) 230 (35)	1.17 (1.06-1.29) 1.08 (0.96-1.21)	1.15 (1.04-1.27) 1.07 (0.95-1.20)	0.34
*GSR*	rs1002149 GG GT+TT	456 (69) 208 (31)	424 (64) 240 (36)	1.06 (0.97-1.17) 1.27 (1.12-1.45)	1.05 (0.96-1.16) 1.24 (1.09-1.42)	0.048
*SOD1*	rs202445 AA AG+GG	436 (66) 228 (34)	463 (70) 201 (30)	1.15 (1.06-1.26) 1.08 (0.94-1.24)	1.14 (1.04-1.25) 1.06 (0.92-1.23)	0.42

IRR: incidence rate ratio.

^a^ 39 case-control pairs were excluded from this analysis due to one participant (or both) in the case-control pair was/(were) abstainer(s).

^b^ Crude.

^c^ Adjusted for parity (parous/nulliparous, number of births, age at first birth), length of school education (low, medium, high), duration of HRT use (years), previous benign breast disease and BMI (kg/m^2^) at baseline.

^d^ *P*-value for interaction for adjusted risk estimates.

**Table 4:** IRR for BC in relation to the studied polymorphisms per increment of 100 g fruit and vegetables per day

Gene	SNP	n_cases_ (%) (n=703)	n_controls_ (%) (n=703)	IRR^a^ (95% CI)	IRR^b^ (95% CI)	P-value^c^
*CAT*	rs1001179 GG GA+AA	408 (58) 295 (42)	409 (58) 294 (42)	1.00 (0.93-1.07) 1.05 (0.98-1.13)	1.00 (0.93-1.07) 1.05 (0.98-1.13)	0.32
*CAT*	rs12270780 GG GA+AA	393 (56) 310 (44)	405 (58) 298 (42)	1.02 (0.95-1.08) 1.04 (0.97-1.12)	1.01 (0.94-1.07) 1.06 (0.98-1.14)	0.33
*CAT*	rs769217 CC CT+TT	454 (65) 249 (35)	460 (65) 243 (35)	1.05 (0.99-1.11) 0.99 (0.91-1.07)	1.05 (0.99-1.12) 0.98 (0.90-1.07)	0.22
*GSR*	rs1002149 GG GT+TT	480 (68) 223 (32)	452 (64) 251 (36)	1.01 (0.95-1.07) 1.06 (0.98-1.15)	1.01 (0.95-1.07) 1.07 (0.99-1.16)	0.21
*SOD1*	rs202445 AA AG+GG	460 (65) 243 (35)	490 (70) 213 (33)	0.99 (0.93-1.05) 1.13 (1.03-1.24)	0.98 (0.93-1.05) 1.13 (1.03-1.25)	0.016

IRR: incidence rate ratio.

^a^ Crude.

^b^ Adjusted for parity (parous/nulliparous, number of births, age at first birth), length of school education (low, medium, high), duration of HRT use (years), previous benign breast disease, alcohol intake (10 g/day) and BMI (kg/m^2^) at baseline.

^c^ *P*-value for interaction for adjusted risk estimates.

**Table 5:** IRR for BC in relation to the studied polymorphisms and smoking status (present/non-smoker)

Gene	SNP	Non-smokers n_cases_/n_controls_	Present smokers n_cases_/n_controls_	Non-smokers IRR^a^ (95% CI)	Present smokers IRR^a^ (95% CI)	Non-smokers IRR^b^ (95% CI)	Present smokers IRR^b^ (95% CI)	P-value^c^
*CAT*	rs1001179 GG GA+AA	264/279 198/160	144/130 97/134	1.00 (ref.) 1.35 (1.03-1.77)	1.19 (0.89-1.61) 0.78 (0.56-1.07)	1.00 (ref.) 1.41 (1.07-1.87)	1.26 (0.92-1.73) 0.83 (0.59-1.17)	0.0015
*CAT*	rs12270780 GG GA+AA	258/251 204/188	135/154 106/110	1.00 (ref.) 1.04 (0.80-1.35)	0.86 (0.64-1.15) 0.93 (0.66-1.30)	1.00 (ref.) 1.02 (0.78-1.33)	0.89 (0.65-1.21) 0.96 (0.67-1.36)	0.80
*CAT*	rs769217 CC CT+TT	306/281 156/158	148/179 93/85	1.00 (ref.) 0.91 (0.69-1.20)	0.75 (0.57-1.00) 1.01 (0.73-1.41)	1.00 (ref.) 0.93 (0.70-1.24)	0.80 (0.59-1.08) 1.05 (0.74-1.50)	0.14
*GSR*	rs1002149 GG GT+TT	322/288 140/151	158/164 83/100	1.00 (ref.) 0.81 (0.61-1.07)	0.84 (0.63-1.11) 0.76 (0.54-1.06)	1.00 (ref.) 0.82 (0.61-1.10)	0.87 (0.65-1.17) 0.81 (0.57-1.16)	0.61
*SOD1*	rs202445 AA AG+GG	314/306 148/133	146/184 95/80	1.00 (ref.) 1.10 (0.83-1.46)	0.80 (0.61-1.04) 1.16 (0.81-1.66)	1.00 (ref.) 1.14 (0.85-1.53)	0.84 (0.63-1.12) 1.24 (0.85-1.81)	0.29

IRR: incidence rate ratio.

^a^ Crude.

^b^ Adjusted for parity (parous/nulliparous, number of births, age at first birth), length of school education (low, medium, high), duration of HRT use (years), previous benign breast disease, alcohol intake (10 g/day) and BMI (kg/m^2^) at baseline.

^c^ P-value for interaction for adjusted risk estimates.

Activities of CAT, SOD and GR enzymes were measured in a subset of the cohort at the time of entry into the DCH cohort (*n*=434 cases and 434 controls). In linear analysis (Table 6), increment in enzyme activity of the three enzymes was associated with statistically non-significant decreased risk of BC (IRR_CAT_=0.89, 95% CI: 0.75-1.06; IRR_SOD_=0.94, 95% CI: 0.79-1.12; IRR_GR_=0.83, 95% CI: 0.68-1.02). However, in the tertile analyses of the enzyme activity (Table 6), only increasing CAT activity exhibited a dose-dependent decrease in BC risk. In Table 7, the correlation between intake of fruit and vegetables, alcohol and smoking, and enzyme activity was investigated. A negative correlation between alcohol intake and GR activity was found (*P*=0.043) (Table 7). Smoking and intake of alcohol were positively correlated with SOD activity with marginally statistical significance (*P*_alcohol_=0.050 and *P*_present smokers_=0.054) (Table 7). None of the investigated lifestyle factors were associated with CAT activity. In Table 8, the relation between the studied genotypes and enzyme activities was examined. *CAT*/rs1001179, *CAT*/rs12270780 and *GSR*/rs1002149 polymorphisms were strongly associated with enzyme activities (*P*≤0.0001 for all three single nucleotide polymorphisms (SNPs)) among both cases and controls, so that enzyme activities increased for each variant allele for *CAT*/rs12270780 and *GSR*/rs1002149, whereas enzyme activities decreased for CAT/rs1001179 for each variant allele (Table 8). *CAT*/rs769217 polymorphism was also associated with enzyme activity (*P*_all_=0.0007) with enzyme activities decreasing for each variant allele, but only among controls (*P*_controls_=0.0007 and *P*_cases_=0.25) (Table 8). These associations were not significantly modified by intake of alcohol, fruits and vegetables, and smoking status (*P*_int_ between 0.26-0.92 (Supplemental Table 1)). We have previously found association between HRT use and GPX activity [39], but in the present study, we did not find indication of association between CAT, SOD or GR enzyme activities and HRT use (Supplemental Table 2). Haplotype analysis of *CAT* polymorphisms revealed that haplotypes encompassing variant alleles of *CAT*/rs1001179 and *CAT*/rs769217, respectively, were functional, and that haplotypes encompassing the variant allele of *CAT*/rs1001179 had the strongest effect on CAT enzyme activity (Table 9).

**Table 6:** Risk of BC in relation to enzyme activity

Enzyme activity (U/g Hb)	Cases (n=375)	Controls (n=375)	IRR (95% CI)^a^	IRR (95% CI)^b^	P-value
	n (%)	n (%)			
CAT activity^c^	13.8 (10.6-17.3)^f^	13.7 (10.5-17.6)^f^	0.93 (0.79-1.10)	0.89 (0.75-1.06)	0.20
CAT activity for tertiles^g^ ≤12.8 >12.8-14.4 >14.4	125 (33) 128 (34) 122 (33)	119 (32) 121 (32) 135 (36)	1.00 (ref.) 0.99 (0.68-1.45) 0.78 (0.52-1.17)	1.00 (ref.) 0.93 (0.63-1.38) 0.72 (0.47-1.10)	0.72 0.13
SOD activity^d^	840 (643-1071)^f^	832 (658-1082)^f^	0.97 (0.82-1.15)	0.94 (0.79-1.12)	0.49
SOD activity for tertiles^g^ ≤795 >795-900 >900	126 (34) 126 (34) 123 (33)	142 (38) 104 (28) 129 (34)	1.00 (ref.) 1.51 (0.99-2.59) 1.10 (0.70-1.74)	1.00 (ref.) 1.51 (0.97-2.35) 1.00 (0.62-1.61)	0.070 0.99
GR activity^e^	11.4 (9.3-14.0)^f^	11.5 (9.5-14.5)^f^	0.83 (0.68-1.01)	0.83 (0.68-1.02)	0.080
GR activity for tertiles^g^ ≤10.8 >10.8-12.2 >12.2	128 (34) 126 (34) 121 (32)	127 (34) 122 (33) 126 (34)	1.00 (ref.) 1.00 (0.69-1.45) 0.95 (0.65-1.37)	1.00 (ref.) 1.01 (0.69-1.47) 0.97 (0.66-1.42)	0.97 0.86

Values are expressed as medians with 5th and 95th percentiles. 59 case-control pairs were excluded because one or both in the matched set had missing data on one or more of the potential confounding variables and/or enzyme activities. IRR: incidence rate ratio.

^a^ Crude estimates.

^b^ The risk estimates for BC are adjusted for parous/nulliparous, number of births, age at first birth, length of school education (low, medium, high), duration of HRT use (years), BMI (kg/m^2^), previous benign breast disease and alcohol intake (10 g/day).

^c^ Estimates are per increment in activity of 2 U/g Hb.

^d^ Estimates are per increment in activity of 100 U/g Hb.

^e^ Estimates are per increment in activity of 2 U/g Hb.

^f^ Median (5-95%) levels of enzyme activity (U/g Hb) for cases and controls.

^g^ Categories are based on tertiles among both cases and controls.

**Table 7:** Associations between erythrocyte enzyme activity and dietary and lifestyle factors

Dietary and lifestyle factors	CAT activity U/g Hb	P-value	SOD activity U/g Hb	P-value	GR activity U/g Hb	P-value
Alcohol, per 10 g/day	+0.029	0.56	−5.70	0.050	−0.071	0.043
Fruit and vegetables, per 100 g/day	−0.021	0.57	+3.51	0.11	−0.033	0.23
Present smokers compared to non-smokers	−0.22	0.17	+18.83	0.054	+0.033	0.78

The table gives the increase or decrease in enzyme activity per dose.

**Table 8:** Associations between the studied genotypes and erythrocyte enzyme activities

Gene	SNP	Change in enzyme activity measured in U/g Hb (95% CI)		
		Cases (n=386)	Controls (n=357)	All (n=743)
*CAT*	rs1001179 GG GA AA *P*_-value_^a^	0 (ref.) -1.40 (−1.50;0.99) -3.13 (−3.91;-2.35) <0.0001	0 (ref.) -1.79 (−2.23;-1.36) -2.94 (−3.85;-2.03) <0.0001	0 (ref.) -1.59 (−1.89;-1.30) -3.05 (−3.64;-2.45) <0.0001
*CAT*	rs12270780 GG GA AA *P*_-value_^a^	0 (ref.) 1.05 (0.62;1.47) 1.82 (1.04;2.59) <0.0001	0 (ref.) 0.93 (0.46;1.40) 1.64 (0.47;2.81) <0.0001	0 (ref.) 0.99 (0.67;1.30) 1.75 (1.10;2.40) <0.0001
*CAT*	rs769217 CC CT TT *P*_-value_^a^	0 (ref.) -0.35 (−0.79;0.10) -0.50 (−1.63;0.62) 0.25	0 (ref.) -0.89 (−1.39;-0.40) -1.35 (−2.72;0.03) 0.0007	0 (ref.) -0.60 (−0.93;-0.26) -0.86 (−1.74;0.01) 0.0007
*GSR*	rs1002149 GG GT TT *P*_-value_^a^	0 (ref.) 1.69 (1.44;1.95) 4.25 (3.49;5.02) <0.0001	0 (ref.) 2.14 (1.85;2.42) 3.83 (3.09;4.56) <0.0001	0 (ref.) 1.90 (1.71;2.09) 4.02 (3.49;4.55) <0.0001
*SOD1*	rs202445 AA AG GG *P*_-value_^a^	0 (ref.) -3.17 (−31.90;25.56) 8.90 (−61.96;79.76) 0.94	0 (ref.) 7.99 (−22.05;38.03) 87.71 (12.86;162.56) 0.071	0 (ref.) 2.16 (−18.60;22.92) 46.70 (−4.75;98.16) 0.21

In this analysis, only individuals with missing values on genotypes and potentials confounders were excluded without regard to the match set.

^a^ P-value for trend.

**Table 9:** Change in CAT enzyme activity in U/g Hb in relation to haplotype combination

Haplotype combination	GGC	AGC	P-value^a^	GAC	P-value^a^	GGT	P-value^a^
GGC	0 (ref.)	−1.8	<0.0001	0.3	0.25	−1.0	0.0004
**A**GC		−3.3	<0.0001	−1.3	<0.0001	−2.7	<0.0001
G**A**C				0.1	0.68	−0.9	0.0030
GG**T**						−1.6	<0.0001

^a^ P-value for comparison to the wild-type haplotype (G-rs1001179A, G-rs12270780A, C-rs769217T). Variant alleles are in bold.

We found an increased risk of BC among women carrying both the low activity A-allele of *CAT*/rs1001179 and high activity G-allele of *SOD1*/rs202445 (IRR: 1.43; 95% CI: 1.01-2.01) (Table 10). The interaction was, however, not significant on a multiplicative scale (*P*_int_=0.27), but could be considered additive. We also investigated other combinations of polymorphisms in relation to risk of BC, but did not find anything further (results not shown).

**Table 10:** IRR for BC for combinations of *CAT*/rs1001179 and *SOD1*/rs202445 genotypes

*CAT*/rs1001179	IRR (95% CI)^a^	IRR (95% CI)^b^			*P*-value^c^
	*SOD1*/rs202445				
	AA	AG+GG	AA	AG+GG	
GG	1.00 (ref.)	1.12 (0.83-1.50)	1.00 (ref.)	1.12 (0.83-1.53)	
GA+AA	0.96 (0.74-1.23)	1.31 (0.94-1.83)	0.98 (0.75-1.26)	1.43 (1.01-2.01)	0.27

IRR: incidence rate ratio.

^a^ Crude.

^b^ Adjusted for parous/nulliparous, number of births, age at first birth, length of school education (low, medium, high), duration of HRT use (years), BMI (kg/m^2^), previous benign breast disease and alcohol intake (10 g/day).

^c^ P-value for interaction on a multiplicative scale.

## DISCUSSION

In the present study, we found that genetically determined variations in the antioxidant enzyme activities of *CAT*, *GSR* and *SOD1* were not associated with risk of BC *per se* in this relatively large cohort. However, intake of alcohol, fruit and vegetables, and smoking status interacted with some of the polymorphisms in relation to BC risk. All polymorphisms, except for *SOD1*/rs202445, were associated with enzyme activity, but there was no interaction between any of the studied environmental factors and polymorphisms in relation to enzyme activity. Haplotype analysis of *CAT* polymorphisms revealed that *CAT*/rs1001179 had the strongest effect on CAT enzyme activity. Additionally, single measurement of enzyme activity at entry to the cohort was not associated with risk of BC.

Both *in vivo* [48–50] and *in vitro* [49] studies have shown that alcohol is capable of decreasing GR activity and protein levels in rat breast [48], liver [49] and brain tissue [50]. The present study supports these findings, since intake of alcohol is associated with decreased overall GR enzyme activity and increased risk of BC among carriers of the high activity T-variant allele of the *GSR*/rs1002149 polymorphism. Carriage of the variant allele of this polymorphism is associated with statistically non-significant decreased risk of BC *per se*, indicating that alcohol may inhibit GR activity in the variant enzyme. However, alcohol did not interact with *GSR*/rs1002149 in relation to enzyme activity in the present study. It is well-known that alcohol consumption increases oxidative stress [51,52] and *in vitro* studies have shown that acetaldehyde (the product of the first step in alcohol oxidation) can interact with GSH spontaneously [53] (forming a GSH-acetaldehyde conjugate). Although this may explain only a minor proportion of observed GSH reduction after acute ethanol intoxication, it is not clear whether such effects are seen at moderate alcohol intake and to what extent this affects GSH availability in breast tissue. A possible inhibitory action of ethanol and/or acetaldehyde on enzymes involved in GSH synthesis has also been hypothesized [48,53]. GSH is critical in maintaining a reduced cellular environment and plays an important role in detoxification processes, including removal of peroxides. Potential GSH depletion combined with impaired GR enzyme activity may impact antioxidant defense, leading to increased carcinogenic potential in the breast tissue. We observed previously that alcohol intake also influences glutathione peroxidase by increasing the activity only in wild-type carriers of the *GPX1* gene and that the *GPX1* Pro198Leu polymorphism may increase BC risk [31]. GSH availability seems therefore potentially important for BC in accordance with the current findings regarding GR.

We were not able to identify other studies examining the relationship between *SOD1* polymorphism, BC risk and potential effect modification by intake of fruit and vegetables. Results from studies investigating the effect of increased intake of fruit and vegetables on SOD activity are inconsistent and the reported effects probably depend both on the presence of specific compounds in the investigated fruit and vegetables as well as the study population. Both negative [54], positive [55,56] and no [23] effects of intake of specific (or combinations of) fruits and vegetables on SOD enzyme activity have been reported. Our results indicate that fruit and vegetables consumption may affect BC risk differently in carriers of the variant allele of *SOD1* polymorphism compared to wild-type carriers. Per 100 g increment in fruit and vegetable intake, risk of BC increased significantly among variant allele carriers only. The reason for this is not clear, but if fruit and vegetables increase SOD activity, this could potentially lead to increased formation of the highly reactive hydroxyl radicals, leading to oxidative damage and increased risk of BC if not metabolized to less reactive compounds. The increased risk of BC observed in carriers of both the high-activity *SOD1* and low-activity *CAT* (rs1001179) allele could support this explanation. However, whether this is a plausible explanation awaits further studies in larger populations with higher intakes of fruit and vegetables.

Our finding regarding the association between *CAT*/rs1001179 and decreased CAT enzyme activity is consistent with two other studies [33,57]. Compared to the other studied *CAT* polymorphisms (*CAT*/rs12270780 and *CAT*/rs769217), *CAT*/rs1001179 is associated with the largest decline in enzyme activity among 743 postmenopausal women in the present study. This is also shown in the haplotype analysis (Table 9) where the variant allele of *CAT*/rs1001179 is only present on the AGC haplotype which is associated with the largest decrease in CAT enzyme activity. Studies on BC risk, however, have mostly failed to find associations between BC and *CAT*/rs1001179 *per se* [reviewed in 54]. In the present study, high CAT enzyme activity was associated with non-significantly lowered BC risk, and variant allele carriers of *CAT*/rs1001179 were at non-significantly increased BC risk.

We found interaction between *CAT*/rs1001179 and smoking status in relation to BC. Among non-smokers, genetically determined lower CAT activity was associated with increased risk of BC, whereas among smokers, homozygous wild-type allele carriers were at non-significantly increased risk. Smoking was though not associated with CAT activity. Taken together, the results may suggest that CAT genetically determined high enzyme activity is protective of BC especially among non-smokers. While smoking had no statistically significant effect on CAT enzyme activity, smoking may change the cellular oxidant/antioxidant environment due to the presence of other compounds in the smoke, thereby masking the effect of the polymorphism we report among non-smokers. Whether the inconsistent effects observed among smokers and non-smokers are linked to the possible anti-estrogenic effect of smoking reported in some studies [59,60] is not clear but needs further investigation.

We used a prospective, nested case-control design, which together with complete follow-up minimizes selection bias. In addition, information on life style factors was collected at enrolment, which reduces the risk for differential misclassification between cases and controls. The study is fairly large to study main effects, it is homogenous and alcohol consumption is relatively high in the DCH cohort [61] making it suitable for studying gene-environment interactions. However, Danes including the Danish women in the present study have a relatively low intake of fruit and vegetables (Table 1), and therefore, the present study may have limited statistical power to detect effects of fruits and vegetables consumption on antioxidant enzyme activities. The genes were carefully selected based on known and predicted functionality. Nevertheless, we are aware that our study may not be large enough for some of the gene environment interactions, although we have previously found gene-environment interactions for *PPARG* and alcohol intake and *ADH* and alcohol intake in relation to BC in the present cohort [40,41].

Neither enzyme activities measured once at study entry nor genetically determined differences in enzyme activities were associated with risk of BC. There are two possible interpretations; either that antioxidant activity is not involved in breast carcinogenesis. Alternatively, genetically determined variations are small compared to the influence of lifestyle factors. However, smoking, alcohol intake and fruit and vegetable intake only correlated with the measured enzyme activities to a very limited extent. Thus, the genetically determined changes in the enzyme activities were larger than the effects of the lifestyle factors studied here. Our results therefore suggest that the antioxidant enzyme activities included here are not major determinants of BC risk.

## MATERIALS AND METHODS

### Subjects

The subjects were selected from the ongoing Danish DCH cohort study. The present study group has been described previously [40,41]. In short, 79,729 women aged 50–64 years, born in Denmark, living in the Copenhagen or Aarhus areas and having no previous cancers at the time of invitation were invited to participate in the study between December 1993 and May 1997. A total of 29,875 women accepted the invitation, corresponding to 37% of the invited women.

Study participants were followed up for diagnosis of BC from date of entry until either the date of diagnosis of cancer using record linkage to the Danish Cancer Registry until 2003 and afterwards by linkage to the Danish Pathology Databank, date of death, date of emigration, or April 27th, 2006, whichever came first. A total of 975 women were diagnosed with BC during the follow-up period. For each case, one matched control was selected [40,41]. The control was cancer-free at the exact age at diagnosis of the case and was further matched on age at inclusion into the cohort (half-year intervals), use of HRT (current/former/never) and on certainty of postmenopausal status (known/probably postmenopausal) upon inclusion into the cohort [40,41]. 208 individuals were excluded because of failed genotyping or no buffy coat was available. Additionally 82 individuals were excluded because of missing information about one or more of the potential confounding variables. 254 individuals were excluded because of a missing partner in the case-control pair, due to the above mentioned exclusions leaving 703 pairs for data analyses.

### Data on covariates

From food frequency and lifestyle questionnaires, we obtained information on duration of school education, smoking status, HRT use, birth pattern (number of births and age at first birth), alcohol intake and diet. Body mass index (BMI) was computed based on measurements of height and weight at enrolment. Intake of alcohol was inferred from the food-frequency questionnaire and life-style questionnaire as described in details in [40,41]. Abstainers were defined as those who reported no intake of alcohol on the food-frequency questionnaire and no drinking occasions on the lifestyle questionnaire. Findings on all known risk factors have been reported previously for both the entire DCH cohort, for a subset of the present study, and for the present study group [40,42–46].

### Blood sampling and storage

From non-fasting participants a total of 30 ml blood was collected in citrated (2 × 10 ml) and plain (1 × 10 ml) Venojects from each participant. Plasma, serum, lymphocytes and erythrocytes were isolated and frozen at −20°C within 2 hours. At the end of the day of collection, all samples were stored in liquid nitrogen, at −150°C.

### Genotyping

DNA was isolated from frozen lymphocytes as described [62]. Generally, 100 mg DNA was obtained from 10^7^ lymphocytes. All polymorphisms were genotyped by KBioscience (KBioscience, Hoddesdon, United Kingdom) by PCR-based KASP™ genotyping assay (http://www.lgcgenomics.com/). To confirm reproducibility, genotyping was repeated for 10 % of the samples yielding 100% identical genotypes.

### Selection of polymorphisms

Common, functional polymorphisms in *SOD1*, *CAT* and *GSR* were chosen with minor allele frequencies between 0.31 and 0.44 which is considered ideal when studying gene-environment interactions [63–65]. Functionality was either based on a literature search or using the http://manticore.niehs.nih.gov/snpinfo/snpfunc.htm website [66], which is a web-based tool that predicts functionality of SNPs. *SOD1*/rs202445 is located in the regulatory region of the promotor [67]. *GSR*/rs1002149 and *CAT*/rs1001179 are also located in the promoter region [58,67] at a transcription factor binding site [66]; and *CAT*/rs1001179 correlates with catalase activity [33,57,68]. *CAT*/rs769217 is located in exon 9 [58] at an exonic splicing site [66]; and *CAT*/rs12270780 is located in intron 2 (http://www.ncbi.nlm.nih.gov/projects/SNP/snp_ref.cgi?rs=12270780) at a transcription factor binding site [66].

### Erythrocyte enzyme activities

In a subset of the present cohort comprising 434 case-control pairs, SOD, CAT and GR activities were determined spectrophotometrically in erythrocyte lysates on a Cobas Mira analyzer (F. Hoffmann-La Roche Ltd, Basel, Switzerland) according to Wheeler et al. [69]. This subset has been described in details elsewhere [31]. Intra- and interday CVs were 2,8% and 4,7% for GR, 6,8% and 9,7% for SOD and 4,6% and 9% for CAT.

### Statistical analyses

Deviation from Hardy-Weinberg equilibrium was assessed using a Chi square test.

Due to the study design using incidence density sampling of controls with match on age at diagnosis, matched logistic regression analyses leads to estimation of BC incidence rate ratio (IRR) [70] corresponding to a Cox proportional hazard model for the full cohort with age as the time axis. The associations between genotype, enzyme activity and BC are presented as crude IRR as well as adjusted for potential BC risk factors. Two-sided 95% confidence intervals (CI) for the IRR were calculated based on Wald's test of the Cox regression parameter, i.e. on the log RR scale. All models were adjusted for baseline values of risk factors for BC such as parity (entered as two variables; parous/nulliparous and number of births), age at first birth, length of school education (low, medium and high), duration of HRT use, alcohol intake and BMI. IRR was calculated separately for heterozygous and homozygous variant allele carriers. For all the SNPs, variant allele carriers were subsequently grouped for interaction analyses to improve the statistical power since no recessive effects were observed. For the different genetic variations, we investigated interactions with alcohol intake, smoking, and fruit and vegetables, using the likelihood ratio test.

A covariance model was used to determine the effects of fruits and vegetables, intake of alcohol and smoking on enzyme activity according to genotype. Enzyme activity was found to be normally distributed among the participants, and hence, the activity was entered untransformed as the dependent variable in the covariate models.

The procedures PHREG (Proportional Hazard Regression) and GLM (General Linear Model) in SAS, release 9.3; SAS Institute, (Cary, NC) were used for the matched logistic regression analyses and the covariance analyses, respectively.

### Ethics statement

All participants gave verbal and written informed consent before enrolment to the study. The DCH study was approved by the regional Ethical Committees on Human Studies in Copenhagen and Aarhus (jr.nr.(KF) 11–037/01), and by the Danish Data Protection Agency.

## SUPPLEMENTARY MATERIALS TABLES



## Abbreviations

BC: breast cancer
BMI: body mass index
CAT: catalase
CI: confidence intervals
DCH cohort: “diet, cancer and health” cohort
GR: glutathione reductase
HRT: hormone replacement therapy
IRR: incidence rate ratio
ROS: reactive oxygen species
SNP: single nucleotide polymorphism
SOD: superoxide dismutase.
