# Supplementary material for: Association between single nucleotide polymorphisms in the antioxidant genes CAT, GR and SOD1, erythrocyte enzyme activities, dietary and life style factors and breast cancer risk in a Danish, prospective cohort study
Source: Oncotarget. 2017 May 22;8(38):62984–97. doi: 10.18632/oncotarget.18062 (PMC5609897; doi:10.18632/oncotarget.18062)
Supplement: Supplementary file 1 [file oncotarget-08-62984-s001.pdf]

Association between single nucleotide polymorphisms in the antioxidant genes *CAT*, *GR* and *SOD1*, erythrocyte enzyme activities, dietary and life style factors and breast cancer risk in a Danish, prospective cohort study

SUPPLEMENTARY MATERIALS

Supplementary Table 1: Genotype specific effects of alcohol intake, fruit and vegetables intake, and current smoking on erythrocyte enzyme activity

Gene	SNP	Change in enzyme activity measured in U/g Hb (95% CI)			
		Per 10 g alcohol/day[b]	Per 100 g fruit and vegetables/day[b]	All non-smokers[c]	All smokers[c]
CAT	rs1001179				
	GG	0.22 (0.12;0.32)	0.16 (0.09;0.23)	0 (ref.)	-0.18 (-0.56;0.20)
	GA	-0.35 (-0.49;-0.21)	-0.17 (-0.25;-0.09)	-1.61 (-1.98;-1.24)	-1.74 (-2.19;-1.29)
	AA	-0.86 (-1.21;-0.50)	-0.41 (-0.54;-0.28)	-2.78 (-3.55;-2.02)	-3.61 (-4.54;-2.69)
	P_{trend}	0.26	0.80	0.54	
	$P_{interaction}{}^{a}$				
CAT	rs12270780				
	GG	-0.11 (-0.22;-0.00)	-0.11 (-0.19;-0.03)	0 (ref.)	-0.21 (-0.62;0.20)
	GA	0.25 (0.12;0.38)	0.07 (-0.01;0.16)	0.97 (0.57;1.37)	0.82 (0.34;1.30)
	AA	0.48 (0.13;0.83)	0.23 (0.07;0.38)	1.93 (1.12;2.74)	1.19 (0.11;2;27)
	P_{trend}	0.49	0.79	0.71	
	$P_{interaction}{}^{a}$				
CAT	rs769217				
	CC	0.12 (-0.00;0.23)	0.01 (-0.07;0.09)	0 (ref.)	-0.38 (-0.78;0.02)
	TC	-0.07 (-0.19;0.06)	-0.08 (-0.17;0.01)	-0.76 (-1.18;-0.35)	-0.68 (-1.18;-0.19)
	TT	-0.12 (-0.59;0.35)	-0.13 (-0.33;0.07)	-1.01 (-2.05;0.03)	-0.98 (-2.58;0.62)
	P_{trend}	0.85	0.28	0.40	
	$P_{interaction}{}^{a}$				
GSR	rs1002149				
	GG	-0.26 (-0.33;-0.19)	-0.16 (-0.20;-0.11)	0 (ref.)	-0.05 (-0.26;0.17)
	TG	0.33 (0.24;0.43)	0.21 (0.15;0.26)	2.03 (1.78;2.27)	1.69 (1.40;1.97)
	TT	1.25 (0.85;1.64)	0.75 (0.60;0.90)	4.05 (3.31;4.80)	3.95 (3.21;4.70)
	P_{trend}	0.54	0.56	0.35	
	$P_{interaction}{}^{a}$				
SOD1	rs202445				
	AA	-6.23 (-12.47;0.02)	2.88 (-1.71;7.46)	0 (ref.)	25.46 (2.47;48.45)
	AG	-6.81 (-15;92;2.30)	4.33 (-1.01;9.67)	8.09 (-18.16;34.35)	17.02
	GG	10.40 (-10,20;30.99)	11.08 (-1.35;23.52)	60.08 (0.86;119;29)	(-14.62;48.65)
	P_{trend}	0.92	0.35	0.62	43.23
	$P_{interaction}{}^{a}$				(-60.13;146.60)

[a] P-value for interaction between genotype and risk factor adjusted for the isolated effect of the genotype and the risk factor.

[b] Among all participants.

[c] All non-smokers compared to all smokers.

Supplementary Table 2: Association between erythrocyte enzyme activity and HRT use

	CAT activity U/g Hb	P-value	SOD1 activity U/g Hb	P-value	GR activity U/g Hb	P-value
Use of HRT, years	+0.019	0.76	-6.53	+0.076	-0.017	0.71

Among ever users of HRT. The table gives the increase or decrease in enzyme activity per additional 5-year of HRT use.
